# Perioperative Cardioprotection by Remote Ischemic Conditioning

**DOI:** 10.3390/ijms20194839

**Published:** 2019-09-29

**Authors:** Youn Joung Cho, Won Ho Kim

**Affiliations:** Department of Anesthesiology and Pain Medicine, Seoul National University Hospital, Seoul National University College of Medicine, Seoul 03080, Korea; mingming7@gmail.com

**Keywords:** remote ischemic conditioning, cardioprotection, neuronal pathway, humoral pathway, cardiac surgery, propofol, diabetes

## Abstract

Remote ischemic conditioning has been investigated for cardioprotection to attenuate myocardial ischemia/reperfusion injury. In this review, we provide a comprehensive overview of the current knowledge of the signal transduction pathways of remote ischemic conditioning according to three stages: Remote stimulus from source organ; protective signal transfer through neuronal and humoral factors; and target organ response, including myocardial response and coronary vascular response. The neuronal and humoral factors interact on three levels, including stimulus, systemic, and target levels. Subsequently, we reviewed the clinical studies evaluating the cardioprotective effect of remote ischemic conditioning. While clinical studies of percutaneous coronary intervention showed relatively consistent protective effects, the majority of multicenter studies of cardiac surgery reported neutral results although there have been several promising initial trials. Failure to translate the protective effects of remote ischemic conditioning into cardiac surgery may be due to the multifactorial etiology of myocardial injury, potential confounding factors of patient age, comorbidities including diabetes, concomitant medications, and the coadministered cardioprotective general anesthetic agents. Given the complexity of signal transfer pathways and confounding factors, further studies should evaluate the multitarget strategies with optimal measures of composite outcomes.

## 1. Background

Myocardial infarction results in tissue damage as a consequence of both ischemia and subsequent reperfusion injury [1]. However, a brief episode of myocardial ischemia and reperfusion can protect the myocardium from prolonged ischemia/reperfusion by activating the molecular defense mechanisms [2]. This protective conditioning of the myocardium can be induced locally by a brief episode of occlusion/reperfusion of the coronary artery. However, remote conditioning is also possible through brief episodes of ischemia/reperfusion of a distant limb: This transfer of protection is called remote ischemic conditioning.

In the original animal studies, repeated brief preceding cycles of occlusion/reperfusion of the left circumflex artery reduced the size of a subsequent myocardial infarct in the left anterior descending artery territory [3]. Initially, this effect was regarded as an incidental finding. However, subsequent studies confirmed the protective effect and found that protection can be transferred from a number of distant tissues and organs [4,5]. Ischemic conditioning is now regarded as a systemic response, which can be triggered by various stimuli in addition to ischemia/reperfusion, and can be elicited at different tissues and organs [6]. Remote ischemic conditioning confers protection before or even after the myocardial infarct-causing ischemia. Remote ischemic conditioning is now categorized as preconditioning (before myocardial ischemia), perconditioning (during myocardial ischemia), or postconditioning (after the target organ ischemia) [7]. Delayed remote conditioning refers to the procedure in which the time interval between conditioning and target ischemia is longer than several minutes [8]. Although an exact dose–response relationship has not been established, previous studies suggested that the frequency and duration of remote ischemic conditioning episodes, rather than the volume of remote ischemic tissue affected, determine the degree of resulting target tissue protection [9,10]. The mechanism by which remote ischemic conditioning transfers the protective signal is largely unclear [11]. Both humoral and neuronal factors seem to be engaged in the transfer, with interactions reported between the two factors [2].

Clinical studies provided evidence of the remote ischemic conditioning effect. Transient episodes of ischemia/reperfusion induced by cycles of inflation and deflation of a blood pressure cuff placed on the upper limb protected the contralateral upper limb from ischemia/reperfusion-induced endothelial dysfunction [12,13]. In a subsequent clinical trial, remote ischemic preconditioning (RIPC) by cycles of ischemia/reperfusion reduced the extent of myocardial injury measured by troponin release during elective coronary artery bypass surgery [4]. A subsequent study has reported that the remote ischemic perconditioning implemented in an ambulance vehicle during the transport of patients with acute myocardial infarction to the hospital reduced myocardial infarct size measured by single-photon emission computed tomography [14]. RIPC was shown to provide protection against acute kidney injury in patients undergoing cardiac surgery [15]. These promising studies elicited many subsequent clinical trials to reproduce the protective effects and laboratory studies to elucidate the mechanism of transfer of protection. 

## 2. Signal Transduction

### 2.1. Remote Stimulus

Remote ischemic conditioning elicits tissue protection as a result of a remote stimulus. In addition to the repeated cycles of ischemia/reperfusion of a limb or organ, a number of remote stimuli were shown to induce protective effects in the target organ. The stimuli include: (i) Brief cycles of ischemia/reperfusion of a limb or organ [16,17,18,19,20]; (ii) mechanical stimuli, including surgical skin incision [21]; (iii) chemical-pharmacologic stimuli, including autacoids (such as adenosine [22], bradykinin [23]), or capsaicin [24]; and (iv) electrical stimuli, including electroacupuncture or percutaneous electrical nerve stimulation [23,24]. As the dose–response relationship has not been established for any of these stimuli, previous comparisons of different stimuli have limited value [19,20]. Peripheral sensory nerve stimulation is involved in all these stimuli. The extent to which peripheral nerve stimulation or additional mechanisms are involved in remote ischemic conditioning, however, is uncertain [6,25].

### 2.2. Signal Transfer—Neuronal and Humoral Factors

Both humoral and neuronal factors are involved in the transfer of protective signals from the site of a remote stimulus to the target organ. However, the exact amount or nature of humoral and neuronal mediators remain uncertain [2,6,26]. Previous studies that investigated neuronal or humoral mediators are summarized in Table 1.

Neuronal factors are engaged in the remote conditioning of the heart [27], as evidenced by a large number of studies suggesting their involvement. Bilateral cervical or subdiaphragmatic vagotomy and cell-specific neuronal silencing in the brain stem nucleus of the vagus nerve were found to abolish the reduction in myocardial infarct size in rats by remote ischemic conditioning [28]. Electrical stimulation of the vagus nerve confers effects similar to remote ischemic conditioning in pigs and rabbits [28,29]. Intrathecal lidocaine attenuated the effect of remote intrathecal morphine preconditioning by blocking spinal cord neuronal signal transmission [30]. Spinal cord transection at the T9–T10 level abrogated the effect of remote conditioning by three episodes of limb ischemia/reperfusion [31]. However, it is still not clear whether the involvement of the brain stem is mandatory for the transduction of the protective signal or which level of the spinal cord is involved in the pathway. A previous study reported that the effect of remote ischemic conditioning could be transferred to distant tissue in non-diabetic patients or diabetic patients without diabetic neuropathy. Conversely, the protection was not transferrable in diabetic patients with peripheral neuropathy, providing further evidence of a neuronal pathway [32].

It is evident that the humoral signal is transferred to the heart from the site of the remote stimuli. However, we do not know whether the same humoral factors underlie the effects elicited by different stimuli. Humoral cardioprotective signals can be identified in the plasma, plasma dialysate, or whole blood of the donor who underwent remote conditioning and can be transferred to a recipient by transfusion [2,33,34,35]. Previous animal studies revealed that the cardioprotection elicited by remote ischemic conditioning can be transferred to an isolated perfused heart of the same or even other species using plasma dialysate, diluted plasma, or whole blood [2,33,35]. However, the efficacy of cardioprotection was not compared between plasma dialysates at different dilutions, or between diluted plasma and whole blood. Investigation of successful humoral transfers have identified two key characteristics of humoral factors: The molecular size of humoral factors is less than 12 to 14 kDa and humoral factors remain effective at plasma dilutions up to 1:50. Humoral factors were further determined to be thermolabile, lyophilizable, and hydrophobic, suggesting that humoral factors are proteins or peptides [35,36,37]. In healthy volunteers who underwent three cycles of forearm ischemia/reperfusion (5 min each), plasma dialysate could be used to transfer cardioprotection even after seven days [34]. A fluctuation in plasma proteins over time was observed, with up/downregulation detected by proteomic analysis of plasma obtained from healthy volunteers who underwent remote ischemic conditioning [38]. However, the factors were not identified, and the causal role of the proteins was not clearly determined.

A microRNA array found an increase in circulating microRNA144 levels following remote ischemic conditioning. Exogenous microRNA144 was shown to induce cardioprotection, while a specific antisense oligonucleotide abrogated the ability of remote ischemic conditioning to reduce infarct size [39]. Elevations in apolipoprotein A1 were identified by proteomic approaches in both human and rat plasma after remote conditioning [38,40]. Levels of endothelial RNase1 were increased in the blood of patients undergoing heart surgery after remote ischemic conditioning, suggesting it could be a humoral mediator [41]. However, a causal relationship was not proven because the troponin level was not reduced by conditioning in this particular study.

Autacoids, such as adenosine or bradykinin, are released during regional ischemic conditioning and exogenous administration of autacoid confers cardioprotection. Adenosine release into the spinal cord tissue was associated with reduced infarct size in response to remote conditioning [42]. Adenosine levels were increased in the arterial plasma after persistent brain ischemia in mice [43]. This increase in adenosine was associated with a subsequent cardioprotective effect and reduction of infarct size in isolated perfused mice hearts [43]. In patients undergoing coronary angiography, exogenous adenosine infusion was associated with cardioprotection, which could be transferred to reduce infarct size in perfused isolated mouse hearts [44]. While it is not considered to be cardioprotective itself, adenosine is believed to stimulate the release of cardioprotective factors. Bradykinin is not regarded as a humoral transfer factor of remote conditioning [45].

Cytokines or chemokines are considered humoral factors involved in the signal transfer pathways. Anti-inflammatory responses were associated with local ischemic conditioning [2]. In mice, plasma concentrations of interleukin-10 (IL-10) were increased after remote ischemic conditioning. Genetic knock-out of IL-10 or IL-10 receptors abolished the infarct size reduction induced by remote conditioning [46]. Stromal cell-derived factor-1α (SDF-1α) release was observed in response to remote ischemic conditioning. Inhibition of SDF-1α chemokine 4 receptors attenuated the effect of remote ischemic conditioning on infarct size [17]. Neuropeptides, including substance P and calcitonin gene-related peptide (CGRP), are considered to be causally involved in the reduction of infarct size. Substance P and CGRP receptor antagonists abrogated the effect of remote conditioning on infarct size [47].

Hormones, including erythropoietin and glucagon-like peptide-1 (GLP-1), were proposed as potential humoral factors. Remote ischemic conditioning in mice increased serum erythropoietin, which was in turn associated with a reduced infarct size. Exogenous administration of erythropoietin antibodies abrogated the reduction in infarct size [48]. In rats, vagus nerve activation following remote ischemic conditioning mediated a release of GLP-1. Infarct size was reduced by GLP-1 agonist. Subdiaphragmatic vagotomy or blockade of M3 muscarinic receptor abolished the effect by the GLP-1 agonist [28]. However, circulating GLP-1 minimally increased following remote conditioning [11]. The active form of GLP-1 cannot be differentiated from its inactive form [49].

Amino acids were proposed to be among the humoral factors involved in remote conditioning. In mice, genetic deletion or pharmacologic inhibition of a *EGLN1* gene product, which converts α-ketoglutarate to succinate in skeletal muscle, was shown to reduce infarct size. It was possible to transfer humoral factors from donor mice with a deletion of the *EGLN1* gene to wild type recipient mice, resulting in a reduction of infarct size following occlusion/reperfusion of the coronary artery [50]. *EGLN1* gene deletion leads to an increase in the expression of hypoxia-inducible factor-1α (HIF-1α) protein [50]. HIF-1α is known as the main regulator of cytokine and hormonal signaling pathways activated following hypoxia [51]. The reduction in infarct size by remote conditioning was abolished in HIF-1α heterozygous knockout mice [52]. *EGLN1* gene inhibition induced by hypoxia could be one upstream regulator of HIF-1α [50]. The amino acids kynurenine and glycine were identified as being associated with remote conditioning. Infarct size was reduced by infusing exogenous kynurenine and glycine in rats prior to myocardial ischemia [53]. However, the causal relationship between kynurenic acid, *EGLN1*, and remote conditioning remains to be established.

In rats, systemic inhibition of nitric oxide synthase abolished the effect of remote conditioning on infarct size [54,55]. However, systemic pretreatment with a nitric oxide synthase inhibitor in rabbits did not attenuate the protective effect of remote ischemic conditioning [20]. Two human studies were performed, aiming to quantify the release of nitric oxide following remote conditioning by measuring the sum of plasma nitrite and nitrate levels. A release of nitrate and nitrite was found following remote conditioning in patients undergoing elective coronary intervention [26], while no such release was observed in healthy volunteers [56].

It is now recognized that the humoral and neuronal factors interact on three distinct steps in remote ischemic conditioning: The remote stimulus, systemic level (involving transfer of signal), and target tissue. On the level of the stimulus, peripheral sensory neuronal pathways are activated, with an associated release of humoral factors and activation of the peripheral nerve [27]. On the systemic level, neuronal signals from peripheral nerves travel to the autonomic nervous centers in the brain and activate the efferent vagus nerve [27]. Vagal stimulation leads to a release of acetylcholine in both target and non-target organs and activates the muscarinic receptors, resulting in a release of humoral factors, such as GLP-1, which subsequently elicits their effects on the target organ [28]. At the target tissue level, the nervous system of the target organ is activated by the humoral factors released by the conditioning [28,57]. Additionally, the responses of parenchymal cells in the target organ are mediated by the humoral factors.

Since protective effects were demonstrated in a number of human organs, the response to remote conditioning is believed to be systemic in nature. Ischemia/reperfusion injury was reduced by remote conditioning in the heart, lung, brain, liver, intestine, kidney, pancreas, and skeletal muscles [58]. Therefore, it can be postulated that common systemic signaling pathways are engaged in remote conditioning. However, the specific signaling pathways involved may also be organ specific. Mitochondria are regarded as common and important mediators, involved in the last steps of the signaling cascades underlying the protective pathways [2,59]. Mitochondria occupy 30% of the cytoplasmic volume of cardiomyocyte [60]. The activities of cytoprotective enzymes, such as catalase and superoxide dismutase, are low in cardiomyocytes [61]. However, signal transduction pathways have not been compared between different organs at the same time. In cardiomyocytes, the intracellular signaling seems to be species specific [2].

### 2.3. Myocardial Response

In the myocardium, protective signaling cascades are apparently activated by incoming neuronal and humoral transfer signals [25]. The cascade is activated in a receptor-independent manner or through the sarcolemmal receptors, with mitochondria believed to be the intracellular target.

The signal transduction pathways in the myocardium affected by remote ischemic conditioning are considered to be similar to the pathways activated by local ischemic conditioning. Neurohormones, such as acetylcholine, opioids, and autacoids, act on G protein-coupled receptors. Signaling cascades that involve protein kinases converge on the cytoskeleton and the mitochondria. Reactive oxygen species also initiate receptor-independent signals modulating cardioprotection. The intracellular signal can be transduced through three steps: (1) The reperfusion injury salvage kinase (RISK) pathway; (2) the endothelial nitric oxide synthase/protein kinase G (eNOS/PKG) pathway; and (3) the survivor activating factor enhancement (SAFE) pathway [2,25].

The upregulation of genes involved in cytoprotection (*Hsp73*) and those involved in the response to oxidative stress (*Fabp4*, *Prdx4*, *Hadhsc*) was detected by genomic profiling of the myocardium in mice 15 min after remote conditioning. Downregulated genes include pro-inflammatory genes, such as *Dusp1*, *Dusp6*, and *Egr-1* [62]. Levels of sarcomeric Z-disk-related phosphoproteins were observed to be increased in mouse myocardium by proteomic profiling following remote ischemic conditioning [63]. In cardiac surgical patients, several cell stress-associated proteins and thioredoxin were upregulated after remote ischemic conditioning [64].

### 2.4. Coronary Vascular Response

Characteristics of ischemia/reperfusion injury of the coronary circulation include endothelial dysfunction, impaired vasomotor function, increased permeability, edema, stasis with intravascular cell aggregates, and microembolization of atherothrombotic debris. Destruction of capillaries due to hemorrhaging is one of the severe consequences of ischemia/reperfusion injury [65]. Therefore, coronary microcirculation may be the target of remote ischemic conditioning. Previous studies demonstrated improvements in coronary flow and microcirculation following ischemia/reperfusion in pigs [66], mice [34], and humans [67]. In healthy volunteers, remote conditioning stimulated nitric oxide synthase activity, improved red blood cell deformability [68], and reduced leukocyte adhesion [69]. Remote ischemic conditioning also reduced monocyte-platelet aggregation in healthy volunteers [70], in patients who underwent catheter ablation for atrial fibrillation [71], and in those who underwent coronary angiography [72].

## 3. Clinical Studies Evaluating Remote Ischemic Conditioning

Since promising expectations on the cardioprotective effect of RIPC have evolved from pre-clinical and proof-of-concept studies, researchers have focused on the clinical benefit of RIPC in patients presenting acute myocardial infarction undergoing coronary intervention or in patients undergoing cardiac surgery subjected to myocardial ischemia-reperfusion injury. Most clinical studies have proven the effects of RIPC on reducing post-procedural release of myocardial injury biomarkers, but RIPC did not elicit a clear benefit in terms of clinical outcomes. However, previous studies cannot completely exclude the impact of various confounding factors related to patient characteristics, concomitant medications, anesthesia, and surgery-related parameters.

### 3.1. Percutaneous Coronary Intervention

A plethora of studies have evaluated the effect of remote ischemic conditioning in patients undergoing cardiac surgery and percutaneous coronary intervention (PCI; Table 2). These investigations were motivated by the fact that cardiac surgery and PCI are exemplary models of myocardial ischemia/reperfusion injury and involve predictable periods of ischemia and restoration of perfusion. The myocardium is subjected to acute ischemia/reperfusion injury during revascularization or on initiation or weaning from cardiopulmonary bypass, upon prevailing left ventricle dysfunction and heart failure. As more patients with advanced age and co-morbidities receive cardiovascular surgery, there are increasing unmet clinical needs for novel myocardial protective strategies that would ensure better prognosis. Although the cardioprotective effects of remote ischemic conditioning have been demonstrated in preliminary animal experiments, there are significant discrepancies between the protective effects reported in pre-clinical and clinical trials, particularly when comparing the surgical conditions to the interventional environment.

In clinical studies conducted in patients with ST-segment elevated myocardial infarction (STEMI) undergoing primary PCI, remote conditioning was shown to elicit a relatively consistent reduction in markers of myocardial injury following the procedure [13,71,72]. A randomized trial reported the protective effect of four cycles of upper limb RIPC in patients with acute myocardial infarction receiving primary PCI before hospital admission [14]. RIPC increased the myocardial salvaged area at 30 days after intervention, as measured by myocardial perfusion imaging. RIPC was also effective when applied on arrival at the hospital before primary PCI [73] and even at the onset of reperfusion during coronary intervention [74].

RIPC also improved patient clinical outcomes after primary coronary intervention [73,74]. In patients undergoing primary PCI, four cycles of 5-min ischemia/reperfusion of the upper limb reduced the incidence of major adverse cardiac and cerebrovascular events as well as all-cause mortality compared to the control group [75]. Three cycles of intermittent ischemia in the lower limb prior to angioplasty in patients presenting with STEMI improved the combined outcome of cardiac mortality and hospitalization for heart failure during the post-procedural 2 years [76]. However, another recently reported large multicenter randomized trial on the effect of RIPC in patients with STEMI undergoing primary PCI revealed no benefit in cardiac death or hospitalization for heart failure at 12 months after PCI, compared to the control arm [77]. RIPC, therefore, seems to have beneficial effects, reducing the release of post-procedural myocardial injury biomarkers, but it is not clear whether RIPC improves clinical outcomes in patients with STEMI undergoing primary PCI. Current European Society of Cardiology guidelines for management for patients with STEMI suggests only a limited benefit of RIPC [78], while the American College of Cardiology/American Heart Association guidelines have no mention of ischemic conditioning in the management of patients with stable ischemic heart disease [79] or STEMI undergoing primary PCI [80].

### 3.2. Cardiac Surgery

Perioperative application of remote ischemic conditioning did not consistently elicit beneficial effects in terms of target organ protection [5,85,92,93,95,96], in contrast to peri-procedural applications, such as primary PCI or catheter ablation [13,71,72]. In the first adult study of cardiac surgery [4], three cycles of 5-min ischemia and 5-min reperfusion of the upper limb using a pressure cuff inflated to 200 mmHg reduced troponin-T release during 48 h after coronary artery bypass graft (CABG) surgery. Several subsequent studies have reported favorable outcomes following this landmark trial. Similarly, RIPC protocol also reduced the postoperative release of cardiac troponin-I within 72 h after CABG and reduced all-cause mortality in the 1.5 years following surgery [5]. Two cycles of simultaneous upper and lower limb ischemia/reperfusion reduced 72-h postoperative troponin-T concentrations in patients undergoing CABG and/or heart valve surgery, as compared to the control group [85]. However, not all studies evaluating RIPC during cardiac surgery demonstrated favorable results and a reduction in cardiac biomarkers. Hong et al. did not observe any reduction in the postoperative release of troponin-I during 72 h following the application of four cycles of RIPC in patients undergoing off-pump CABG [82]. In patients with concentric myocardial hypertrophy undergoing aortic valve replacement, three cycles of upper limb RIPC did not affect the 24-h area under the curve for creatine kinase-myocardial band or troponin-T levels following the surgery [88]. Although some inconsistent results have been presented, a recent meta-analysis reported the protective effects of RIPC, as measured by cardiac biomarkers. After pooling the results of 30 trials of CABG or valve surgery, RIPC was found to reduce postoperative troponin release compared to the control arm [97]. Moreover, RIPC ameliorated sinus rhythm restoration up to 1 year after surgery and reduced postoperative markers of systemic inflammation in patients undergoing Cox maze radiofrequency ablation with concomitant mitral valve surgery [98].

However, despite the reported protective effects on the surrogate markers of myocardial injury, several large-scale multicenter studies did not find any beneficial effect of RIPC on clinical outcomes, including the rate of major adverse cardiac events and mortality, as compared to the control group [92,93,95,96]. Given these negative clinical studies, a recent Cochrane review concluded that there is no evidence of a treatment effect of RIPC on clinical outcomes when assessed as a composite endpoint (including all-cause mortality, myocardial infarction, or stroke) in patients undergoing CABG and/or valve surgery, although RIPC did reduce postoperative troponin-T release at 72 h after surgery [99]. A currently available position paper by the European Society of Cardiology Joint Working Group reported that RIPC may attenuate perioperative myocardial injury without a significant benefit on clinical outcomes in patients undergoing CABG surgery [100], but the American College of Cardiology/American Heart Association guideline did not mention it in the management of patients with valvular heart disease [101].

Unsuccessful translation of the protective effects observed in studies in healthy animals into clinical trials was attributed to the underlying risk factors present in patients and to the concomitant use of potentially cardioprotective agents [102]. Notably, the use of the anesthetic agent propofol, which was used in the majority of patients in the aforementioned phase III trials [92,93,95], was proposed to be a major confounding factor, potentially interrupting the RIPC process [5,87,103,104]. During CABG surgery, three cycles of RIPC in the upper limb reduced postoperative release of cardiac troponin-I under isoflurane/sufentanil anesthesia but did not have any effect when used in propofol/sufentanil anesthesia in patients with ischemic heart disease [103]. Propofol was suggested to abrogate the cardioprotective effect of RIPC in anesthetized patients. A previous study demonstrated that propofol interferes with the transduction of the cardioprotective signal of RIPC in patients with ischemic heart disease by inhibiting the phosphorylation of signal transducer and activator of transcription (STAT5) [84]. In a subsequent randomized controlled study where propofol was excluded from the anesthetic regimen, RIPC reduced postoperative troponin I release during 72 h post-surgery, and a decreased rate of all-cause and cardiac mortality was observed during the 1.5-year follow-up [5]. This beneficial effect was sustained in the long-term analysis, with patients followed up for a median duration of 5 year (up to 9 year) following CABG surgery [104]. In small randomized trials in patients undergoing on- [89] or off-pump CABG surgery [90] without propofol, RIPC was reported to reduce postoperative release of cardiac troponins.

Volatile anesthetic agents were also suggested to exhibit protective effects and their use could be a confounding factor in the evaluation of the effect of RIPC. This organ protective effect is supported by a meta-analysis that showed a beneficial effect of volatile anesthesia on survival after cardiac surgery by pooling the data from 38 trials after excluding studies with ischemic or remote ischemic conditioning [105]. A Bayesian network meta-analysis of 55 trials also demonstrated the beneficial effect of RIPC during cardiac surgery on short- and long-term survival with the use of volatile anesthetic agents [106]. Xie et al. reported a possible protective effect of volatile anesthetic agents based on a meta-analysis of 30 trials evaluating a total population of 7036 patients undergoing cardiac surgery. They concluded that RIPC significantly reduced postoperative markers of myocardial injury but did not reduce the incidence of acute myocardial infarction or acute kidney injury, and had no effect on mortality [97]. However, RIPC reduced the incidence of acute kidney injury and mortality in the subset of patients who were anesthetized using volatile agents.

However, in these two meta-analyses [82,93], the propofol group was defined to include only patients who were never exposed to any volatile agent during the surgery, while the volatile group included patients with any exposure to volatile agents, regardless of the dose and timing, even if added to a regimen involving propofol. Therefore, it is difficult to completely exclude the potential residual effect of propofol in the volatile group in these analyses. Considering all the previous study results and limitations, the Cochrane review could not conclusively confirm or rule out propofol as a confounding factor in the assessment of RIPC effectiveness in cardiac surgical patients, mainly due to the limited number of studies and the lack of a control for confounding variables, such as co-morbidities and concomitant-medications [99,107]. Moreover, these findings should be interpreted with caution, considering the risk of bias, including the number of patients who received each anesthetic [108].

A recent study by Behmenburg et al. explored the influence of various anesthetic regimens used during RIPC on myocardial protective effects in an in vivo animal study [109]. The authors compared three anesthetic regimens (pentobarbital alone, sevoflurane combined with remifentanil, and propofol combined with remifentanil) on the effect of four cycles of bilateral hind limb RIPC followed by induction of left anterior descending regional myocardial ischemia/reperfusion injury. Myocardial protective effects of RIPC were preserved under pentobarbital- and sevoflurane-based anesthesia, while the effect of RIPC was abrogated during propofol anesthesia. A subsequent randomized clinical trial compared sevoflurane and propofol, the two most frequently used anesthetics, combined with continuous infusion of remifentanil in patients undergoing cardiac surgery and receiving four cycles of upper limb RIPC [110]. However, in that study, both anesthetics hindered the myocardial protective effects of limb RIPC, whereas the control group that received RIPC preoperatively in an awake state and underwent cardiac surgery did show a reduction in infarct size in the rat myocardium perfused with dialysate from the preconditioned patients. This study considered patient factors (such as comorbidities and concomitant medications), the effect of general anesthesia-related interference on the neural signaling pathway, and the concentration of propofol infused during the study period as confounding factors.

### 3.3. Other Potential Confounding Factors for the Effect of RIPC

Other confounding factors that may contribute to the no effects of RIPC on cardioprotection in cardiac surgery patients include nitrates [75,98] and beta-adrenergic blocking agents [110,111,112,113,114]. However, a retrospective analysis of a randomized trial did not find any significant associations between age, gender, beta-adrenergic receptor blocking agents, angiotensin converting enzyme inhibitors, angiotensin receptor blockers, intraoperative nitroglycerine, and RIPC-induced cardioprotection during CABG [115]. For patients undergoing PCI, prior episodes of angina or ischemia, door-to-balloon time, and any interaction or delay between them would also be important confounders.

The age of subjects receiving RIPC could also be a major confounding factor. Compared to the young adult rat, the heart of a middle-aged rat exhibited a blunted response to ischemic conditioning, while the aged rat heart could not be preconditioned through mechanical or pharmacological methods [116]. One major difference between experimental conditions and clinical trials is the age of subjects receiving RIPC. Response to ischemic conditioning in aging hearts or elderly patients was reported to be resistant to the protective effect of RIPC in several previous studies [117,118,119]. RIPC reduced the length of ICU stay and ventilation time, as well as postoperative serum troponin T levels in children (mean age of 11 months) undergoing heart surgery for congenital defects using cardiopulmonary bypass [120]. In this study, the anesthetic agents used included sevoflurane, midazolam, fentanyl, and sufentanil but not propofol. In contrast to these reports, a post-hoc subgroup analysis of a randomized controlled trial showed that the cardioprotective effect of RIPC was not different between patients older or younger than 70 years [119]. However, this post-hoc analysis had a limited sample size of only 139 patients, putting the study power in question.

Moreover, disappointing results were obtained from the analysis of RIPC effects on mitigating ischemia/reperfusion injury in patients with type 2 diabetes, suggesting the potential role of diabetes as a confounding factor [118,121]. RIPC failed to reduce infarct size in obese rats with type 2 diabetes compared to lean rats [122]. An underlying cause for this effect could involve the association of diabetes with impaired humoral communication of cardioprotective signals in cardiomyocytes subjected to hypoxia-reoxygenation [122]. Diabetes also modifies the myocardial response to ischemic conditioning and interrupts intracellular signaling, thus potentially obscuring the cardioprotective effects of ischemic conditioning [121].

Treatment with anti-diabetic agents can also impact on the cardioprotective action of ischemic conditioning. Blood glucose-lowering agents can modulate intracellular signaling pathways within the heart, which may modulate the protective efficacy of RIPC [123]. Since 2011, the U.S. Food and Drug Administration has recommended that companies developing new drugs for type 2 diabetes provide the evidence that the drug will not increase the risk of cardiovascular events. Since then, most of the new anti-diabetic drugs have favorable aspects regarding myocardial protection [124]. However, the effect of a RIPC-lowering postoperative cardiac biomarker was abolished in sulphonylurea-treated diabetic patients undergoing coronary artery surgery under isoflurane anesthesia [125].

Diabetic neuropathy may impair the effect of RIPC through a suspected interruption in the neuronal pathways [32]. While there was no transferrable protective effect of RIPC from patients with diabetic peripheral neuropathy, the protective effect was transferrable from non-diabetic subjects or diabetics without peripheral neuropathy. Therefore, it has been suggested that RIPC should be repeated over a prolonged period (several days to weeks) to overcome the unresponsiveness of diabetic subjects to ischemic conditioning [126].

Other confounders during cardiac surgery include the use and duration of cardioplegia, which may affect the extent of myocardial injury and might also impact on signal transduction pathways [127]. Hypothermia, advances in intraoperative myocardial protective strategies, and improvement of surgical techniques could be confounding factors because they may reduce perioperative myocardial injury as well as postoperative mortality [7]. Moreover, cross-clamp versus all bypasses during one clamp, types of cardioplegic solutions, and delayed or prolonged weaning from CPB also can be important confounders during cardiac surgery, which remain as subjects for further investigation.

### 3.4. Future Study Directions

Recently, failure of the translation of pre-clinical experimental effects into clinical patient outcomes has been attributed to several newly discovered key factors. Multi-target and multi-strategic trial designs are suggested as potential approaches to overcome the multifaceted nature of myocardial ischemia/reperfusion injury [128,129]. Although myocardial injury biomarkers are surrogates for serious morbidity and mortality after cardiac surgery and PCI, clinical outcomes are multifactorial and attributable not only to regional or global ischemia/reperfusion injury but more substantially to underlying comorbidities, perioperative complications, inconsistent procedural strategies, and large inter-individual variability.

The ultimate goal of any cardioprotective strategy is to improve clinical outcomes. However, appropriate measures of clinical outcomes are challenging due to variations in study conditions. Consequently, cardioprotective effects of RIPC should be evaluated using multitarget strategies and optimized measures of composite outcomes in future studies considering the complex clinical circumstances of cardiac surgical patients.

## Figures and Tables

**Table 1:** Studies on the humoral and neuronal mediators of remote ischemic conditioning.

Study	Design	Animal Type	Mediator	Stimulus/Intervention	Effect
Neuronal factors					
Basalay 2016 [28]	Animal	Rats	Vagus nerve	Vagotomy	Abolished RIC effect
Donato et al., 2013 [31]	Animal	Rabbits	Spinal cord	Transection at T9–10 level	Abolished RIC effect
Donato et al., 2013 [31] Uitterdijk et al., 2015 [29]	Animal	Pig and rabbits	Vagus nerve	Electrical stimulation	Effects similar to RIC
Lu et al., 2014 [30]	Animal	Rats	Spinal cord	Intrathecal lidocaine	Abolished intrathecal morphine preconditioning effect
Humoral factors					
Basalay 2016 [28]	Animal	Rats	GLP-1	Blockade of GLP-1 receptors	Abolished cardioprotection by RIC
Hildebrandt et al., 2016 [34]	Human to animal	Healthy volunteers, mouse heart	Unspecified	Plasma dialysate	Could transfer cardioprotection, STAT3 is activated I murine myocardium.
Li et al., 2014 [39]	Animal	Mice	MicroRNA144	Exogenous administration	Induced cardioprotection
Hepponstall et al., 2012 [38]	Human	Healthy volunteers	Apolipoprotein A1	Following RIC	Elevated in human plasma by RIC
Hibert et al., 2013 [40]	Animal	Rat	Apolipoprotein A1	Following RIC	Elevated in rat plasma by RIC
Cabrera-Fuentes et al., 2015 [41]	Human	Patients undergoing heart surgery	Endothelial RNase 1	Following RIC	Elevated after RIC
Mei et al., 2017 [42]	Animal	Rats	adenosine or bradykinin	Exogenous administration	Confers cardioprotection
Schulte et al., 2004 [43]	Animal	Mice	adenosine	Brain ischemic conditioning	Adenosine increase by mice brain ischemia could transfer protection to mice heart
Contractor 2016 et al. [44]	Animal	Mice	adenosine	Exogenous administration	Associated with cardioprotection
Pedersen et al., 2011 [45]	Human	Healthy volunteers	Bradykinin	Bradykinin receptor antagonist	Had no effect on the protection by RIC
Cai et al., 2012 [46]	Animal	Mice	IL-10	Genetic knock-out of IL-10 or IL-10 receptor antibodies	Abolished infarct size reduction by RIC
Davidson et al., 2013 [16]	Animal	Rats	SDF-1α	Inhibiting SDF-1α chemokine 4 receptor	Attenuated the infarct size-reducing effect of RIC
Gao et al., 2007 [47]	Animal	Rats	Substance P, CGRP	Antagonist of substance P or CGRP	Abrogated the infarct size reduction
Oba et al., 2015 [48]	Animal	Mice	Erythropoietin	Exogenous administration of erythropoietin antibodies	Abrogated the infarct size reduction
Olenchock et al., 2016 [50]	Animal	Mice	*EGLN1* gene	*EGLN1* gene deletion	reduced the infarct size and decreased expression of HIF-1α.
Cai et al., 2013 [52]	Animal	Mice	HIF-1α	HIF-1α knockout	Abolished the infarct size reduction effect
Chao de la Barca et al., 2016 [53]	Animal	Rats	kynurenine and glycine	Exogenous administration	Reduced myocardial infarct size
Donato et al., 2016 [54], Shahid et al., 2008 [55]	Animal	Rats	nitric oxide	Inhibition of systemic nitric oxide synthase	Abolished the effect of RIC to reduce infarct size
Steensrud et al., 2010 [20]	Animal	Rabbits	adenosine	Femoral artery infusion of adenosine	Reduced myocardial infarct size
			nitric oxide	Systemic nitric oxide synthase inhibitor	Did not reduce the protective effect of RIC
Arroyo-Martinez et al., 2016 [26]	Human	Patients undergoing PCI	Nitrate and nitrite	Following RIC	Nitrate was released into coronary artery blood
Lambert et al., 2016 [56]	Human	Human volunteers	Nitrate and nitrite	Following RIC	No release

RIC, remote ischemic conditioning; GLP-1, glucagon-like peptide-1; STAT, signal transducer and activator of transcription; IL-10, interleukin-10; SDF-1α, stromal cell-derived factor-1α; CGRP, calcitonin gene-related peptide, HIF-1α, hypoxia-inducible factor-1α, PCI, percutaneous coronary intervention.

**Table 2:** Clinical studies on the cardioprotective effect of remote ischemic preconditioning in patients undergoing cardiac surgery.

Study	Number, RIPC/Control	RIPC Protocol			Surgey	Anesthetic Induction/Maintenance	Results
		I/R Cycles	Cuff Pressure	Limb			
Cardiac biomarkers							
Hausenloy et al., 2007 [4]	27/30	3 × 5 min	200 mmHg	Upper	CABG	Midazolam, etomidate, propofol/isoflurane, propofol	Reduced TnT during 72 h
Venugopal et al., 2009 [81]	23/22	3 × 5 min	200 mmHg	Upper	CABG ± aortic valve surgery	Midazolam, etomidate, propofol/volatile, propofol	Reduced 72-h AUC for TnT
Hong et al., 2010 [82]	65/65	4 × 5 min	200 mmHg	Upper	Off-pump CABG	Midazolam/sevoflurane	No difference in TnI during 72 h
Young et al., 2012 [83]	48/48	3 × 5 min	200 mmHg	Upper	High-risk cardiac surgery	Midazolam/propofol, isoflurane	No difference in 6-h and 12-h TnT
Thielmann et al., 2013 [5]	162/167	3 × 5 min	200 mmHg	Upper	CABG	Isoflurane or propofol	Reduced 72-h AUC for TnI
Kottenberg et al., 2014 [84]	12/12	3 × 5 min	200 mmHg	Upper	CABG	Etomidate/propofol	No difference in 72-h AUC for TnI
Candilio et al., 2015 [85]	90/90	3 × 5 min	200 mmHg	Upper + lower	CABG ± valve surgery	Midazolam, etomidate, propofol/isoflurane, sevoflurane, propofol	Reduced 72-h AUC for TnT
Walsh et al., 2016 [86]	128/130	3 × 5 min	300 mmHg	Lower	Cardiac surgery	Volatile, propofol	No difference in 24-h CK-MB
Pinaud et al., 2016 [87]	50/49	3 × 5 min	200 mmHg	Upper	AVR	Propofol/isoflurane, sevoflurane, propofol	No difference in 72-h AUC for TnI
Song et al., 2017 [88]	36/36	3 × 5 min	300 mmHg	Upper	AVR	Midazolam/sevoflurane	No difference in 24-h AUC for CK-MB and TnT
Zadeh et al., 2017 [89]	14/14	3 × 5 min	200 mmHg	Upper	CABG	Midazolam, ketamine/isoflurane	Reduced 6-h and 24-h TnI
Wang et al., 2019 [90]	33/32	4 × 5 min	SBP + 40 mmHg	Upper	Off-pump CABG	Midazolam, etomidate/sevoflurane	Reduced 120-h TnT
Jin et al., 2019 [91]	121/120	2 × 5 min	200 mmHg	Upper + lower	Valve replacement surgery	Imidazole valium, propofol	Reduced 6-h and 24-h post-CPB TnT
Clinical outcomes							
Thielmann et al., 2013 [5]	162/167	3 × 5 min	200 mmHg	Upper	CABG	Isoflurane or propofol	Reduced all-cause mortality at 1.5 y
Candilio et al., 2015 [85]	90/90	3 × 5 min	200 mmHg	Upper + lower	CABG ± valve surgery	Midazolam, etomidate, propofol / isoflurane, sevoflurane, propofol	Reduced AKI, AF, and length of ICU stay
Meybohm et al., 2015 [92]	692/693	4 × 5 min	200 mmHg	Upper	Cardiac surgery	Propofol	No difference in composite outcome
Hausenloy et al., 2015 [93]	801/811	4 × 5 min	200 mmHg	Upper	CABG ± valve surgery	Volatile, propofol	No difference in composite outcome
Coverdale et al., 2018 [94]	213/215	3 × 5 min	200 mmHg	Upper	High-risk cardiovascular surgery	Midazolam, propofol, etomidate / desflurane, sevoflurane, propofol	No difference in composite outcome
Jin et al., 2019 [91]	121/120	2 × 5 min	200 mmHg	Upper + lower	Valve replacement surgery	Imidazole valium, propofol	Reduced acute lung injury, and length of ICU and hospital stay

AF, atrial fibrillation; AKI, acute kidney injury; AUC: area under curve; AVR, aortic valve replacement; CABG, coronary artery bypass graft; CK-MB, creatine kinase-myocardial band; ICU, intensive care unit; I/R, ischemia/reperfusion; RIPC, remote ischemic preconditioning; TnI, troponin I; TnT, troponin T.

## Abbreviations

AF	Atrial fibrillation
AKI	Acute Kidney Injury
AUC	Area under curve
AVR	Aortic valve replacement
CABG	Coronary artery bypass graft
CGRP	Calcitonin gene-related peptide
CK-MB	Creatine kinase-myocardial band
eNOS	Endothelial nitric oxide synthase
GLP-1	Glucagon-Like Peptide-1
HIF-1α	Hypoxia-Inducible Factor-1α
ICU	Intensive care unit
I/R	Ischemia/reperfusion
PCI	Percutaneous Coronary Intervention
PKG	Protein Kinase G
RIPC	Remote Ischemic Preconditioning
SAFE	Survivor Activating Factor Enhancement
SDF-1α	Stromal Cell-Derived Factor-1α
STAT-5	Signal Transducer and Activator of transcription-5
STEMI	ST-segment Elevated Myocardial Infarction
TnI	Troponin I
TnT	Troponin T
